# Comparative Phytochemical Analysis and Antioxidant Activities of Tamalakyadi Decoction with Its Modified Dosage Forms

**DOI:** 10.1155/2019/6037137

**Published:** 2019-05-02

**Authors:** Jeevani Maheshika Dahanayake, Pathirage Kamal Perera, Priyadarshani Galappatty, Hettiarachchige Dona Sachindra Melshandi Perera, Liyanage Dona Ashanthi Menuka Arawwawala

**Affiliations:** ^1^Department Ayurveda Pharmacology and Pharmaceutics, Institute of Indigenous Medicine, University of Colombo, Sri Lanka; ^2^Department of Pharmacology, Faculty of Medicine, University of Colombo, Sri Lanka; ^3^Research and Development Complex, Industrial Technology Institute, Malabe, Sri Lanka

## Abstract

**Background and Objective:**

Tamalakyadi decoction (TD) is a classical formulation mentioned in authentic traditional medicine text Sarasankshepaya under nasal diseases and used as a remedy for allergic rhinitis. It consists of 12 plant ingredients. Decoction preparations are widely used in Sri Lankan traditional system and considered effective and safe for treating many disorders. However, decoctions have to be used only in fresh state due to shorter shelf life and loss of stability. This decoction preparation method leads to decreasing the patient compliance and is also time consuming. Hence, the objective of the present study was to convert TD to consumer friendly novel dosage form, namely, freeze dried, spray dried, and traditional ganasara forms.

**Methodology:**

Therefore, we compared the phytochemical constituents and antioxidant activities of TD with its modified dosage forms. The chemical comparison of four dosage forms comprises phytochemical screening, TLC and HPTLC fingerprint profiles and the antioxidant activities by DPPH free radical scavenging activity, Ferric reducing antioxidant power (FRAP), total polyphenol content (TPC), and total flavonoid content (TFC).

**Results:**

Phytochemical screening revealed the presence of alkaloids, saponins, tannins, steroids, flavonoids, phenols, and terpenoids in all dosage forms. However, the saponins, alkaloids, flavonoids, terpenoids, and steroids were more prominent in TD and freeze dried preparation than the other two preparations. HPTLC fingerprint pattern of freeze dried dosage was more similar with HPTLC fingerprint pattern of TD in terms of number of peaks and their intensity compared to that of spray dried and ganasara dosage forms. Antioxidant activities such as DPPH, FRAP, TPC, and TFC were higher in decoction and freeze dried preparation than in spray dried and ganasara preparation.

**Conclusion:**

Freeze dried TD is the most suitable ready to use preparation having similar chemical properties and antioxidant activities to TD.

## 1. Introduction

Tamalakyadi decoction (TD) is an effective herbal decoction used for allergic rhinitis since long time. It is mentioned in authentic traditional medicine text, Sarasankshepaya, under nasa roga (nasal diseases) [1]. Allergic rhinitis is an IgE mediate immune response of the nasal mucosa against inhaled allergens and defined as symptoms of sneezing, rhinorrhea, nasal congestion, and itching of the nose and eyes. It is commonly defined as seasonal or perennial, depending upon whether symptoms are manifested at defined yearly intervals or throughout the year, respectively [2]. This condition is the most common allergic disorder and the prevalence of allergic rhinitis is estimated in the range from 9 % to 42 % [3]. The symptoms of allergic rhinitis may significantly affect a patient's quality of life and can be associated with conditions such as fatigue, headache, cognitive impairment, and sleep disturbances. Appropriate management of allergic rhinitis is an important component in effective management of coexisting or complicated respiratory conditions such as asthma, sinusitis, and sleep apnea [4].

In Ayurveda system of medicine, allergic rhinitis is described as Apeenasa or Peenasa and the concept of allergy is explained under “Asatmyaja vyadhi” (allergic disorders), while its effects are explained in hereditary, Viruddhahara (incompatible foods) and Dushivisha (polluted substances or allergic agents) and Ritu sandhi (seasonal changes) [5].

Effective therapeutic methods for allergic rhinitis including internal as well as external treatments are described in Sri Lankan traditional system of medicine and in Ayurveda medicine. TD is one of the effective decoctions used for allergic rhinitis. It includes 12 ingredients which are mentioned in Table 1. Among those 12 ingredients,* Clerodendrum serratum *(L.) Moon plant and* Solanum indicum *L. plant were replaced by* Premna herbacea* Roxb. and* Solanum melongena* L. plants, respectively, for many years.

Decoction is a basic Ayurveda dosage form which is one of the most commonly used and considered as very effective dosage form in system of traditional medicine. However, the decoction preparations have some drawbacks such as unpleasant taste and have to be used only in fresh state due to loss of stability [6]. Therefore, patients on treatments with decoctions need to prepare it every day which causes difficulties in their busy lifestyles. Hence in this study an approach was made to prepare ready to use user friendly decoction powder by using novel technology and choose the most similar ready to use preparation when compared with the decoction in terms of phytochemical constituents and antioxidant activity.

## 2. Materials and Methods

### 2.1. Collection and Authentication of Plant Materials

The plants used in TD were collected from Colombo city (6° 55' 54.98” N x 79° 50' 52.01” E) Western province, Sri Lanka, between July and August 2018 and authenticated by the Curator of National Herbarium of Peradeniya, Sri Lanka. The contaminants of the raw materials were removed manually, washed with water, and shade dried. Then dried raw materials were crushed to a coarse powder separately and stored in tightly closed containers.

### 2.2. Preparation Method of Tamalakyadi Decoction (TD)

TD was prepared according to the traditional decoction preparation method [7]. Five grams was taken from each ingredient of the formulation and boiled with 1920 ml of water under mild flame to reduce the volume up to 240 ml. Then the decoction was filtered through a single folded cotton cloth and collected to a separate vessel. Same procedure was repeated for eight times and pooled decoction (240 ml ×8) was divided equally (240 ml × 2) into four portions. The fist portion was labeled as TD and others were subjected to prepare modified dosage forms.

### 2.3. Preparation of Freeze Dried Form of Tamalakyadi Decoction (FDF-TD)

TD (240 ml × 2) was freeze dried using a freeze dryer (Telstar LyoBeta) with the temperature – 45°C to 40°C and kept in a refrigerator (at 4°C) until used.

### 2.4. Preparation of Spray Dried Form of Tamalakyadi Decoction (SDF-TD)

TD (240 ml × 2) was spray dried using a spray dryer (Mini Spray drier B-290 BUCHI) with 180°C inlet temperature, 102°C outlet temperature, and 50 kg of feed pressure.

### 2.5. Preparation of Ganasara Form of Tamalakyadi Decoction (GSF-TD)

TD (240 ml × 2) was subjected to mild heat, converted to semisolid form, and oven dried (at 105°C) to prepare the GSF-TD [8].

### 2.6. Phytochemical Screening

Phytochemical screening was carried out according to the methods described by Goveas [9] and Joanne and coworkers [10] with some modifications. In brief, freshly prepared TD (240 ml × 2) and the FDF-TD, SDF-TD, and GSF-TD samples dissolved in hot water (240 ml × 2) separately were subjected to phytochemical screening studies as follows.

### 2.7. Test for Saponins

Five milliliters of extract and 2.5 ml of water were added to a test tube, shaken vigorously, and kept for 10 minutes. Then the froth was mixed with 3 drops of olive oil and shaken vigorously and the formation of emulsion was observed. The presence of stable froth indicates that saponins are found in the extract.

### 2.8. Tests for Tannins

- Ferric chloride test: five drops of Fecl_3_ was added to each extract and mixed well. Appearance of a black precipitate indicates the presence of tannins.
- Lead acetate test: three drops of Pb(OAc)_2_ was added to 5 ml of extract and mixed well. Formation of a yellow precipitate is indicative of tannins.
- Vanillin test: few drops of 10 % vanillin in ethyl alcohol and conc. HCl were added to each extract and mixed well. Appearance of red color indicates the presence of tannins.

### 2.9. Test for Phenols

- Vanillin test: few drops of 10 % vanillin in ethyl alcohol and conc. HCl were added to 2 ml of extract. Appearance of red color indicates the presence of phenols.
- Lead acetate test: three drops of Pb(OAc)_2_ was added to 5 ml of extract and mixed well. Formation of yellow precipitate indicates the presence of phenols.

### 2.10. Test for Alkaloids

- Picric acid test: few drops of picric acid was added to 5 ml of extract and mixed well. Formation of a yellow color crystalline precipitate indicates the presence of alkaloids.
- Tannic acid test: few drops of tannic was added to 5 ml of extract and mixed well. Formation of a yellow color crystalline precipitate indicates the presence of alkaloids.
- Wagner reagent test: two drops of Wagner reagent was added to 2 ml of extract and mixed well. Appearance of a reddish color indicates the presence of alkaloids.

### 2.11. Test for Flavonoids

- Five milliliters of dilute ammonia solution was added to 5 ml of extract followed by the addition of conc. H_2_SO_4_. Appearance of yellow color indicates the presence of flavonoids.
- Five milliliters of extract was added to a test tube containing piece of metallic mg and 3 drops of conc. HCl and heated. Flavonoids give a red-orange color.

### 2.12. Test for Terpenoids

- Salkowski test: extract (5 ml) was mixed with 2 ml of chloroform in a test tube and 3 ml of conc. H_2_SO_4_ was added along the sides of the test tube. Formation of reddish brown color is an indicative of presence of terpenoids.
- Test for sesquiterpenes: one milliliter of conc. H_2_SO_4_ was added to 2 ml of extract and mixed well. A reddish brown color indicates the presence of terpenoids.

### 2.13. Test for Steroids

- Five milliliters of acetic anhydride and 5 ml of conc. H_2_SO_4_ were added to the 5 ml of extract and mixed well. A color change from violet to blue or green color indicates the presence of steroids.
- Lieberman Burchard test reaction: two milliliters of acetic anhydride and 2 ml of conc. H_2_SO_4_ were added to 2 ml of extract and mixed well. Formation of a dark bluish green color indicates the presence of steroids.

### 2.14. Test for Cardiac Glycosides

One milliliter of glacial acetic acid was added to 3 ml of extract and con. H_2_SO_4_ acid was introduced to the bottom of the tube. A reddish brown or violet brown ring at the interface of the two liquids indicates the presence of cardiac glycosides.

### 2.15. Development of Thin Layer Chromatography (TLC) and High Performance Thin Layer Chromatography (HPTLC) Fingerprints

Freshly prepared TD (100 ml) and FDF-TD, SDF-TD and GSF-TD dosage forms dissolved in hot water (100 ml from each) were added separately to a separating funnel containing 50 ml of dichloromethane, mixed well and kept for 20 min. After that, dichloromethane layer was separated. This was done thrice and collected dichloromethane fractions were pooled and evaporated to dryness. Dried dichloromethane fractions of TD, FDF-TD, SDF-TD, and GSF-TD dosage forms were redissolved in 5 ml of dichloromethane separately and spotted on a TLC plate. TLC fingerprint profile was developed for all fractions using dichloromethane, ethyl acetate, and cyclohexane in a ratio of 3:0.5:1.5 v/v. The plate was visualized under UV radiation (both 254 nm and 366 nm) and HPTLC fingerprint patterns were observed by using CAMAG - HPTLC scanner.

### 2.16. Extracts for In Vitro Antioxidant Assays

The powders obtained from freeze drying, spray drying, and ganasara methods were dissolved in methanol to prepare methanolic extracts. Liquid form decoction was dried by evaporation using rotary evaporator and redissolved in methanol.

### 2.17. Antioxidants Assay

The antioxidant activities of these four preparations were assessed by using DPPH free radical scavenging activity, Ferric reducing antioxidant power (FRAP), total polyphenol content (TPC), and total flavonoid content (TFC).

### 2.18. DPPH Free Radical Scavenging Activity

The DPPH free radical scavenging assay was performed according to the method described by Blois, [11] with some modifications in 96-well microplates. Reaction mixture of 200 *μ*l, containing 150 *μ*l of DPPH solution and 50 *μ*l of each extract (dissolved in methanol) of decoction or freeze dried or spray dried or ganasara was incubated at room temperature (25 ± 2°C) for 10 minutes in dark and the absorbance was recorded at 517 nm. Five different concentrations of Trolox (2.5, 5, 10, 20, 30 *μ*g/ml) were used to construct the standard curve. Results were expressed as IC_50_; *μ*g/ml.

### 2.19. Ferric Reducing Antioxidant Power (FRAP)

The assay was carried out according to the Benzie and Strain [12] with some modifications in 96-well microplates. The working FRAP reagent was prepared by mixing 300 mM acetate buffer (pH 3.6), 10 mM TPTZ solution and 20 mM FeCl_3_.6H_2_O (10:1:1 v/v/v) just before use and incubated at 37°C for 8 minutes. Reaction mixtures of 200 *μ*l containing 150 *μ*l FRAP reagent, 30 *μ*l of acetate buffer, and 20 *μ*l of four extracts (120 *μ*g/ml) were incubated at room temperature (25 ± 2°C) for 8 minutes and the absorbance was recorded at 600 nm. Six different concentrations of Trolox (10.3125, 20.625, 41.25, 83.5, 167 *μ*g /ml) were used to construct the standard curve. Results were expressed as mg TE/g of extract.

### 2.20. Total Polyphenol Content (TPC)

Total polyphenol content of four extracts was determined by the Folin-Ciocalteu spectrophotometric method adopted from Singleton and Rossi [13] by using gallic acid as standard phenolic compound using 96-well microplates. Twenty microliters of four extracts, each dissolved in distilled water (150 *μ*g /ml), were added to 110 *μ*l of ten times diluted freshly prepared Folin-Ciocalteu reagent and incubated with 70 *μ*l of 10 % sodium carbonate solution at room temperature (25 ± 2°C) for 30 minutes and the absorbance was recorded at 765 nm. Five different concentrations of gallic acid (0.78, 1.562, 3.125, 6.25, 12.5, 25, and 50 mg/ml) were used to construct the standard curve. Total Polyphenol Content was expressed as mg Gallic Acid Equivalents (GAF)/g of extract.

### 2.21. Total Flavonoid Content of (TFC)

Total flavonoid content of four samples was determined by Aluminium chloride method [14]. One hundred microliters of 2 % Aluminium chloride in methanol solution was incubated with 100 *μ*l of four samples dissolved in methanol (120 *μ*g/ml) at room temperature (25 ± 2°C) for 10 minutes and absorbance was recorded at 415 nm. Six different concentrations of Quercetin (1, 2, 4, 8, 16, 32 *μ*g/ml) were used to construct the standard curve. Total Flavonoid Content was expressed as mg Quercetin Equivalents (QE)/g of extract.

### 2.22. Statistical Analysis

All the assays were performed four times and the absorbance was presented as Mean ± SEM. Analysis of variance was performed using SPSS procedures. The level of significance was used for comparison at 0.05 levels. SPSS* t* – test was used for testing significance level between in other.

## 3. Results and Discussion

In Ayurveda system of medicine we can identify various medicinal preparations mentioned under Bhaishajya Kalpana [15]. Decoctions (kashaya), vati (pills), powders (churna), oils (taila), and arishta-asava (fermented preparations) are few examples for them. These drug preparations can be classified into two: primary preparations and secondary preparations. Panchavidha Kashaya Kalpana is considered as primary preparations which include five types of liquid preparations that are therapeutically effective. These primary preparations are commonly used as the initial dosage forms in treatment and as the base for the different medicinal preparations.

Decoction is one of the effective dosage forms widely used in Ayurveda treatment and the shelf life of this preparation is 24 hours, which means, in the treatment, patient should prepare the decoction everyday [16]. If we are able to develop novel products from decoctions having long shelf life, that would be convenient for people. However, in order to fulfill this requirement, potency of the preparation should be same as the traditional formulation. Potency of a medicine is critical for its efficacy. When modifying the preparation to an easy to use dosage form with appropriate shelf life, active principles or phytochemicals of the drug have to be protected as the traditional preparation.

In this study qualitative phytochemical analysis was done to detect and compare the chemical constituents of TD and its modified dosage forms. Most of the phytochemicals including saponins, alkaloids, flavonoids, phenols, terpenoids, tannins, and steroids were present in all four types of preparations (Table 2). However, saponins, alkaloids, flavonoids, terpenoids, and steroids were more prominent in both traditional TD and FDF-TD than the SDF-TD and GSF-TD. Plant secondary metabolites such as phenols, flavonoids, tannins, and saponins are responsible for many activities including antioxidants, anti-inflammatory, antibacterial, antiasthmatic, immunomodulatory actions etc. [17]. Prolonged administration of saponin from* Clerodendrum serratum *plant has been reported to exhibit antihistaminic and antiallergic activity [18, 19].* C. serratum* is one of the ingredients in TD and high content of saponins was found in both TD and FDF-TD. This factor helps to prove the effectiveness of TD and FDF-TD in the treatment of allergic rhinitis which is characterized by nasal congestion, watery nasal discharge, itching of the nose, and sneezing [20]. Therefore, the above properties of the drug could overcome the symptoms of allergic rhinitis. Further this is the first attempt taking place to screen possible phytochemicals present in TD.

**Figure 1:**
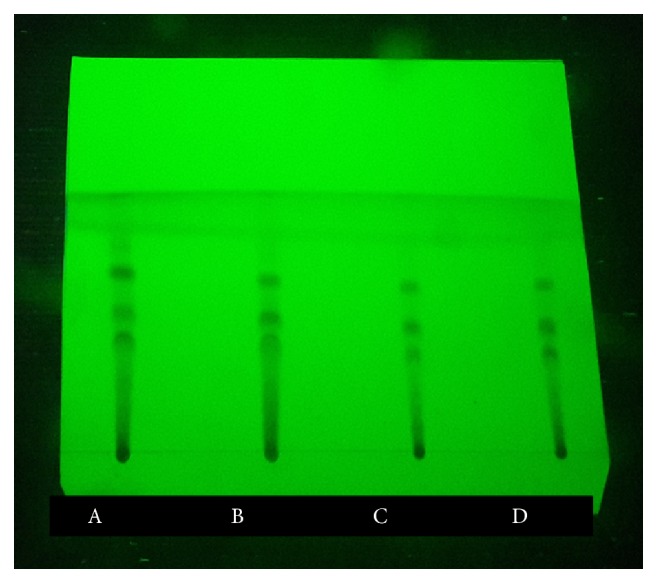
TLC fingerprint profiles of Tamalakyadi Decoction and its modified dosage forms. A: Tamalakyadi decoction (TD), B: Freeze Dried Form of Tamalakyadi Decoction (FDF-TD), C: Spray Dried Form of Tamalakyadi Decoction (SDF-TD), D: Ganasara Form of Tamalakyadi Decoction (GSF-TD).

TLC and HPTLC techniques are used for quality assessment in Ayurvedic preparations. These methods are widely employed in pharmaceutical industry in process of identification, development and quality control of herbal products [21]. However, HPTLC technique is more advanced than TLC and used for quantification purpose. When considering the TLC fingerprint patterns almost similar TLC profiles were observed in all four dosage forms bearing R_f_ values of 0.12, 0.32, 0.43, 0.59, 0.70, and 0.93 (at 245 nm). However, one additional spot was observed in TD bearing R_f_ value of 0.26 (at 366 nm) (Figure 1).

**Figure 2:**
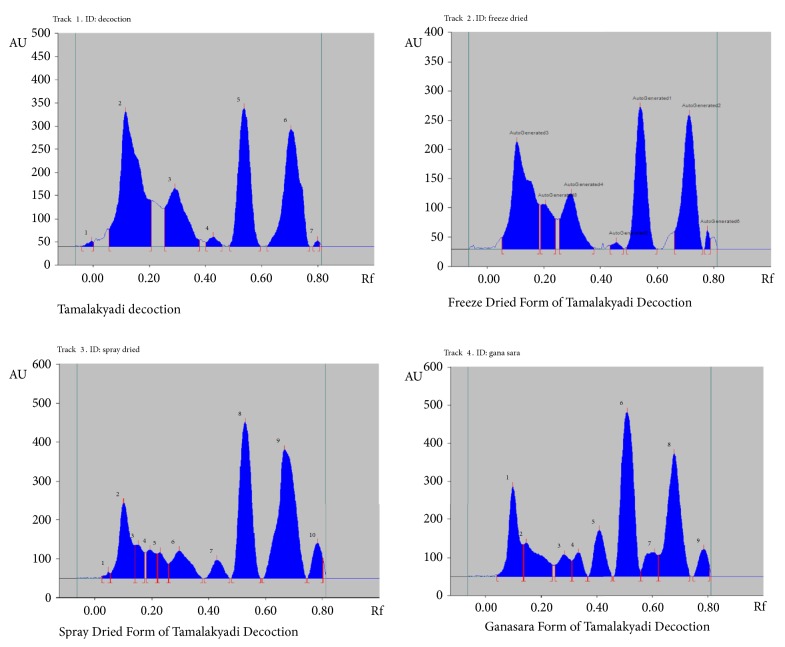
HPTLC fingerprint profiles of Tamalakyadi decoction and its modified dosage forms.

HPTLC study was carried out to compare the area and intensity of the spots appeared in TLC profiles of four preparations. HPTLC fingerprint pattern of TD was similar to that of FDF-TD in terms of number of peaks and their intensity compared to that of SDF-TD and GSF-TD (Figure 2). This may be due to the temperature and time which affect chemical constituents of plant materials during drug preparation. Decomposition of chemical constituents or change in chemical structure or reduction of chemical constituents occurred when increasing the temperature and time [22–26]. TD is the traditional preparation and all the modified dosage forms are made out of it. Therefore, FDF-TD, SDF-TD, and GSF-TD initially subjected to 105°C. However, FDF-TD will not be exposed more than 105°C as we used the freeze drying process while SDF-TD will be exposed to 180°C during the preparation of modifies dosage form. When preparation of GSF-TD will not be exposed more than 105°C but it has to keep prolong time in 105°C. Therefore, heat labile compound/s in both SDF-TD and GSF-TD may be decomposed during the preparation of modified dosage forms. This may be the reason that the chemical profile of FDF-TD was similar to that of TD. In contrast, research findings of Singh and coworkers [27] showed that spray dried form of Lodhradi Kashaya was chemically similar to that of conventional dosage form which was prepared according to the classical method mentioned in Sharangadhara Samhita. Different temperatures used in spray drying in different studies may have accounted for this. The freeze dried dosage form is exposed to low temperature (- 40°C to 40°C) which may cause less damage to phytoconstituents. Research reports revealed, when compared to air drying/oven drying methods, freeze drying improved the retention of phytochemicals during processing and in some cases it even increased the concentration of phytochemicals blue berry and raspberry [28, 29].

Antioxidants are compounds that inhibit or delay onset of oxidation and may be classified as natural or synthetic [30]. There is an increasing demand for natural antioxidants for curing and prevention of diseases indicating that compounds in natural formulations are more active than their isolated form [31]. During the recent years many changes have occurred in the management of allergic rhinitis by using medicines in various traditional medicinal systems [32, 33]. A number of scientific investigations have proven the association between antioxidants and allergic diseases and antioxidant intake seems to have a protective effect on allergic diseases like rhinitis [34–36]. Hence in this study we had examined the antioxidant activities of four preparations to detect most similar antioxidant activity with the TD (Table 3). TD exhibited highest antioxidant activity in terms of capability of scavenging DPPH radicals, ferric reducing antioxidant power, and total phenolic and flavonoid contents (Table 3). Further, among the modified dosage forms, FDF-TD showed the best antioxidant activity and more closer to that of TD. The reason for the reduction of antioxidant activities of SDF-TD and GSF-TD may be due to the temperature used in the drug processing. Recent research findings highlighted that the temperature had an influence on antioxidant activities of the plant materials and degradation of heat sensitive compounds reported to be minimized at the low temperature [37]. Further, the temperature had an effect on total flavonoids and phenolic contents of the plant which have major contribution to the radical scavenging activity [38–40]. Among the four preparations, total flavonoids and phenolic contents are high in TD and therefore it indicated more DPPH radical scavenging activity than the three modified preparations. Also antioxidant assay results showed that the FRAP value is highest in FDF-TD. Similar results were observed in freeze dried samples of leaves and berries of* Cayratia trifolia* [41] and spearmint leaves [42]. This may be due to the formation of ice crystals within the tissue matrix during the freeze drying process which can rupture the cell structure, which allows the exit of cellular components and the access of solvent [43]. Hence it can perform more antioxidant potency in the media.

## 4. Conclusion

Phytochemical studies, HPTLC patterns, and antioxidant studies showed that the FDF- TD is more similar to TD which was prepared according to traditional method. Therefore, FDF-TD can be used as a novel dosage form to treat allergic rhinitis. However, clinical evaluation is needed for further confirmation.

## Figures and Tables

**Table 1:** Ingredients of Tamalakyadi decoction.

	Plant name	Family	Sinhala name used in Sri Lanka	Sanskrit name	Used part
1	*Phyllanthus niruri *L.	Phyllanthaceae	Pitawakka	Tamalaki	Whole plant
2	*Terminalia chebula* Retz.	Combretaceae	Aralu	Haritaki	Fruit cover
3	*Premna herbacea *Roxb.	Lamiaceae	Siritekku	Bharangi	Roots
4	*Piper retrofractum *Vahl	Piperaceae	Siviya	Chavya	Roots
5	*Piper longum* L.	Piperaceae	Tippili	Pippali	Fruits
6	*Solanum trilobatum* L.	Solanaceae	Wel Tibbatu	Vallikantakarika	Whole plant
7	*Tinospora cordifolia* (Thunb.) Miers	Menispermaceae	Rasakinda	Guduchi	Stem
8	*Zingiber officinale* Roscoe	Zingiberaceae	Inguru	Shunti	Dried Rhizome
9	*Piper nigrum* L.	Piperaceae,	Gammiris	Maricha	Fruits
10	*Solanum melongena* L.	Solanaceae	Elabatu	Vruhati	Roots
11	*Solanum xanthocarpum* L.	Solanaceae	Katuwelbatu	Kantakari	Whole plant
12	*Justicia adhatoda* L.	Acanthaceae	Adathoda	Vasa	Whole plant

**Table 2:** Phytochemical screening of Tamalakyadi decoction and its modified dosage forms.

Phyto constituents	Test	TD	FDF-TD	SDF-TD	GSF-TD
Saponins	Frothing test	+++ High	+++ High	+ Present	++ Moderate

Tannins	FeCl_3_ test	+++ (Blue black precipitate)	+++ (Blue black precipitate)	+++ (Blue black precipitate)	+++ (Blue black precipitate)

	Vanillin test	Negative			

	Pb(OAc)_2_ test	+++ (Yellow precipitate)	++ (Yellow precipitate)	+++ (Yellow precipitate)	++ (Yellow precipitate)

Phenols	Vanillin test	Negative			

	Pb(OAc)_2_ test	+++ (Yellow precipitate)	++ (Yellow precipitate)	+++ (Yellow precipitate)	++ (Yellow precipitate)

Alkaloids	Tannic acid test	++ (yellow precipitate)	++ (yellow precipitate)	+ (yellow precipitate)	++ (yellow precipitate)

	Picric acid test	+++ (yellow precipitate)	+++ (yellow precipitate)	+ (yellow precipitate)	++ (yellow precipitate)

	Wagner test	+++ (red colour)	+++ (red colour)	++ (red colour)	++ (red colour)

Flavonoids	Test (a)	+++ (Yellow colour)	+++ (Yellow colour)	++ (Yellow colour)	++ (Yellow colour)

	Test (b)	++ (Orange colour)	+++ (Orange colour)	+ (Orange colour)	++ (Orange colour)

Terpenoids	Salkowski test	+++ (reddish brown colour)	+++ (reddish brown colour)	++ (reddish brown colour)	++ (reddish brown colour)

	Sesquiterpenes test	++ (reddish brown colour)	+++ (reddish brown colour)	+ (reddish brown colour)	++ (reddish brown colour)

Steroids	Test (a)	+++ (violet colour)	+++ (violet colour)	++ (violet colour)	++ (violet colour)

	Liebermann Burchard Test	+++ (Dark bluish green colour)	+++ (Dark bluish green colour)	++ (Dark bluish green colour)	++ (Dark bluish green colour)

Cardiac glycosides		Reddish brown ring formed	Reddish brown ring formed	Reddish brown ring formed	Reddish brown ring formed

-ve: negative, +: positive in low level, ++: positive in moderate level, +++: positive in high level.

*TD*: Tamalakyadi Decoction, *FDF-TD*: Freeze Dried Form of Tamalakyadi Decoction, *SDF-TD*: Spray Dried Form of Tamalakyadi Decoction, *GSF-TD*: Ganasara Form of Tamalakyadi Decoction.

**Table 3:** Antioxidant activities of Tamalakyadi decoction and its modified dosage forms.

Extract	DPPH free radical scavenging activity (IC_50_; *μ*g/mL)	FRAP (mg TE/g of extract)	TPC (mg GAE/g of extract)	TFC (mg QE/g of extract)
Tamalakyadi Decoction	8.2 ± 0.1^a^	572.5 ± 2.3^aa^	206.0 ± 2.3^aa1^	8.2 ± 0.5^aa2^

Freeze Dried Form of Tamalakyadi Decoction	10.6 ± 0.3^b^	634.2 ± 1.3^bb^	148.2 ± 0.7^bb1^	6.2 ± 0.3^bb2^

Spray Dried Form of Tamalakyadi Decoction	20.8 ± 0.3^c^	154.8 ± 1.9^cc^	63.8 ± 2.0^cc1^	2.9 ± 0.1^cc2^

Ganasara Form of Tamalakyadi Decoction	17.9 ± 0.6^d^	222.4 ± 1.0^dd^	69.5 ± 0.4^dd1^	2.9 ± 0.1^dd2^

Trolox (standard)	5.35 ± 0.25^e^	-	-	-

Results are presented as Mean±SEM (n=4).

Values with the different scripts are significantly different P≤ 0.05 from each other.

*TE*: Trolox Equivalents, *GAE*: Gallic Acid Equivalents, *QE*: Quercetin Equivalents.

## Data Availability

The data used to support the findings of this study are available from the corresponding author upon request.
